# Tetranectin, a potential novel diagnostic biomarker of heart failure, is expressed within the myocardium and associates with cardiac fibrosis

**DOI:** 10.1038/s41598-020-64558-4

**Published:** 2020-05-05

**Authors:** Kenneth McDonald, Nadezhda Glezeva, Patrick Collier, James O’Reilly, Eoin O’Connell, Isaac Tea, Adam Russell-Hallinan, Claire Tonry, Steve Pennington, Joe Gallagher, Mark Ledwidge, John Baugh, Chris J. Watson

**Affiliations:** 1 Heart Failure Unit, St Vincent’s University Hospital Healthcare Group, Elm Park, Dublin Ireland; 2 Conway Institute of Biomolecular and Biomedical Research, School of Medicine, University College Dublin, Dublin, Ireland; 3 Department of Cardiovascular Medicine, Cleveland Clinic, Cleveland, Ohio 44195 USA; 4 Wellcome-Wolfson Institute for Experimental Medicine, Queen’s University Belfast, Northern Ireland, UK

**Keywords:** Diagnostic markers, Heart failure

## Abstract

Heart failure (HF) screening strategies require biomarkers to predict disease manifestation to aid HF surveillance and management programmes. The aim of this study was to validate a previous proteomics discovery programme that identified Tetranectin as a potential HF biomarker candidate based on expression level changes in asymptomatic patients at future risk for HF development. The initial study consisted of 132 patients, comprising of HF (n = 40), no-HF controls (n = 60), and cardiac surgery patients (n = 32). Serum samples were quantified for circulating levels of Tetranectin and a panel of circulating fibro-inflammatory markers. Cardiac tissue served as a resource to investigate the relationship between cardiac Tetranectin levels and fibrosis and inflammation within the myocardium. An independent cohort of 224 patients with or without HF was used to validate serum Tetranectin levels. Results show that circulating Tetranectin levels are significantly reduced in HF patients (p < 0.0001), and are associated with HF more closely than B-type natriuretic peptide (AUC = 0.97 versus 0.84, p = 0.011). Serum Tetranectin negatively correlated with circulating fibrosis markers, whereas cardiac tissue Tetranectin correlated positively with fibrotic genes and protein within the myocardium. In conclusion, we report for the first time that Tetranectin is a promising HF biomarker candidate linked with fibrotic processes within the myocardium.

## Introduction

Heart failure (HF) is a major public health problem and will directly impact the lives of approximately 2% of the population, extending to up to 10% within the over 65 year old demographic^1–4^. A major component of HF disease management is diagnosis and monitoring of disease progression and there is increasing application of biomarker tools such as B-type natriuretic peptide (BNP)^5^. One biomarker discovery approach that we previously adopted was to examine the coronary sinus proteome of asymptomatic hypertensive patients with high and low risk of future development of HF, based on their BNP levels^6^. Numerous biomarkers of interest were identified in this discovery study that may be further investigated to determine their value as a single biomarker or in combination with BNP. Herein we describe a protein with limited known function called Tetranectin that was significantly reduced in coronary sinus serum of asymptomatic patients with elevated BNP. Tetranectin, gene name CLEC3B, is a calcium-binding homotrimeric protein from the C-type lectin family of proteins. It is primarily found in serum and in the extracellular matrix (ECM) during development, tissue regeneration and cancer, with low levels in normal adult tissue^7,8^. Tetranectin potentially has an important role in tissue remodelling due to its ability to bind ECM components (fibrin, plasminogen), stimulate proteolytic activation of proteases and growth factors, and regulate ECM proteolysis. It has been suggested to have a protective function within the muscle, bone, and the circulatory system. Serum concentrations of Tetranectin decrease in pathological conditions such as cancer, inflammatory diseases, and coronary artery disease^9–16^. Tetranectin knockout (null) mice have shown to develop features consistent with Parkinson’s disease when aged, and to have impaired fracture and wound healing processes;^17–19^ however data from a cardiac phenotype in these mice is lacking.

Tetranectin was identified in our biomarker discovery study and given previous limited literature we hypothesised that it could be associated with cardiac remodelling and HF. To investigate this, we studied Tetranectin expression in human cardiac tissue and examined serum Tetranectin levels in at-risk non-HF patients and patients with established HF.

## Methods

### Patient recruitment

This study utilised three different patient cohorts with a total population of 356. Firstly, 100 patients were recruited from the Chronic Cardiovascular Disease Management department at St Vincent’s University Hospital, Dublin. This cohort was the validation cohort. Sixty patients were included from the STOP-HF population^20^, who are asymptomatic and have at least one cardiovascular risk factor for the future development of heart failure, as well as 40 stable HF patients from the Heart Failure Unit. To be eligible for inclusion in the STOP-HF no heart failure (no-HF) population, patients must have been deemed asymptomatic following assessment at the time of presentation by an experienced attending cardiologist and were required to have had an ejection fraction ≥50% on echocardiography. To be eligible for inclusion in the HF population, patients were required to have had a hospitalisation for proven New York Heart Association (NYHA) Class IV heart failure (confirmed by an attending cardiologist), continued symptoms of at least NYHA Class II heart failure, and left ventricular ejection fraction ≥50% with Doppler abnormalities of diastolic dysfunction but no evidence of significant valvular heart disease.

The second cohort, the re-validation cohort, consisted of additional 224 patients with heart failure (n = 60) or risk factors for heart failure risk (hypertension and/or diabetes) (n = 164). This additional heart failure cohort was used for the mass spectrometry study.

The third cohort consisted of 32 patients undergoing elective cardiac surgery for coronary artery by-pass grafting or valve replacement at the Cardiology Department, Blackrock Clinic, Dublin. Right atrial appendage tissue was collected from this cohort. All subjects gave written informed consent to participate in this study. The Ethics Committee at St Vincent’s University Hospital approved all study protocols which conformed to the principles of the Helsinki Declaration.

### Clinical and biochemical assessments

Study patients underwent a full physical examination, and phlebotomy was performed by a blinded observer. Physical examination included assessment of waist circumference, body mass index (BMI) calculation, and a heart rate and blood pressure measurement. Peripheral venous blood samples were analysed for B-type natriuretic peptide (BNP, Triage Meter Point of care BNP assay, Biosite Inc.). All patients had electrocardiograms (ECGs) and Doppler echocardiographic assessment. Left ventricular mass was calculated using the Devereux method and was indexed to body surface area. Left atrial volume was calculated using the biplane area length method and was also indexed to body surface area. Left ventricular filling pressures were non-invasively assessed by E/e′ with tissue Doppler measurements taken at the lateral mitral annulus.

### Peripheral blood sampling

Peripheral venous blood samples were obtained at the time of clinical assessment, 24 hours before cardiac surgery. Serum samples were obtained following centrifugation at 2500 g for 10 min at 4 °C. Samples were aliquoted and stored at –80 °C until required. Each serum sample underwent no more than three freeze–thaw cycles prior to its use in enzyme-linked immunosorbent assays (ELISA), radioimmuno assays (RIA), and mass spectrometry (SRM).

### Assessment of patient peripheral serum Tetranectin levels by Mass Spectrometry

Full details of SRM assay are included in the supplementary methods.

Pooled patient serum samples from the re-validation cohort (n = 224) were used for development of the SRM method. Samples were depleted of the 14 most abundant proteins using Multiple Affinity Removal LC (MARS) [Hu-14 column, 4.6×100 mm, Agilent Technologies], according to the manufacturer’s instructions. Both crude and depleted samples were digested with trypsin and de-salted using C18 resin ZipTips® (Millipore) prior to SRM analysis.

Skyline (MacCoss laboratory, Washington DC version 1.4) and Spectrum Mill Peptide Selector (Agilent Technologies, version 3.3.078) software were both used for selection of suitable proteotypic peptides with properties compatible with detection by mass spectrometry. No fewer than 2 peptides were selected for each protein. To aid in method development, synthetic crude peptides were purchased from Thermo Fisher Scientific (PEPotecTM SRM unmodified peptide in plate). Spectral libraries were generated using raw data generated from LC-MS/MS analysis of crude synthetic peptides on an Agilent 6460 Q-TOF mass spectrometer. Five transitions per peptide, with the highest MS signals in the available MS spectral libraries, were used for the initial SRM development. SRM analysis was carried out on a nanoflow reverse phase C18 chromatographic Chip Cube based separation coupled to an Agilent 6460 triple quadrupole mass spectrometer (QqQ). In order to prevent carry over between samples, ‘blank’ samples (buffer only) were loaded at the start and end of each SRM run and between each sample injection. In order to maintain reproducibility and confirm optimum instrument performance, Pierce TM Peptide Retention Time Calibration Mixture (Thermo Scientific) was used as a ‘quality control’ (QC) standard. This QC was loaded to a final concentration of 250 fmol/µl at the beginning, middle and end of each of the SRM runs in order to monitor and normalise QqQ performance between samples and over time.

Data analysis was performed using both Qualitative Mass Hunter Software (Agilent, V 3.3.078) and Skyline (MacCoss lab, V 4.1). Using this software, peak quality and signal intensity was recorded for each measured peptide as well as the quality of the overall total ion chromatogram (TIC).

### Assessment of patient peripheral serum biomarkers by ELISA and RIA

Peripheral serum levels of interleukin (IL) 6, IL8, monocyte chemotactic protein 1 (MCP1), tumor necrosis factor alpha (TNFα), high sensitivity C-reactive protein (hsCRP), matrix metalloproteinase (MMP) 2, MMP9, and tissue inhibitor of metalloproteinases 1 (TIMP1) in the atrial appendage patient cohort were analysed using the electro-chemiluminescent ultrasensitive assay kits (MSD). For each of the inflammatory markers (IL6, IL8, MCP1, TNFα, hsCRP) the assay sensitivity of was <0.7 pg/ml; for MMP2 – assay sensitivity was 120 pg/ml, for MMP9 – 99 pg/ml, and for TIMP1 – 17.8 pg/ml. Signal detection was performed with SECTOR Imager 2400 (MSD). Procollagen I C-terminal propeptide (PICP) (assay sensitivity 2.0 ng/ml) was measured using ELISA (Takara Biochemicals). Procollagen type I N-terminal propeptide (PINP) (assay sensitivity 13.0 ng/ml), type III procollagen peptide (PIIINP) (assay sensitivity 1.9 ng/ml), and C-telopeptide for type I collagen (CITP) (assay sensitivity 0.5 ng/ml) were measured using radioimmunoassay (Orion Diagnostica). The intra-assay variations for determining PINP, PIIINP, and CITP were 7%, <5%, and <8%, respectively. Assays were performed according to manufacturer’s protocol or as previously described^21–23^. Peripheral serum Tetranectin levels in surgical patients, and in the non-HF vs. HF patient cohort were measured with a human Tetranectin/CLEC3B ELISA kit (Biorbyt).

###  Tissue handling, quantitative real-time PCR, and histological staining

Right atrial appendage tissue was collected from 32 patients with ischemic heart disease and/or valvular heart disease undergoing coronary artery bypass surgery or valve replacement surgery (study cohort 3). Immediately after biopsy collection, samples were divided into two parts with one part used for RNA isolation and another part for histological staining.

#### Tissue RNA isolation, reverse transcription, and Quantitative real-time PCR

Tissues (30-35 mg) were stored in Allprotect Tissue Reagent (Qiagen), homogenised using an Ultra Turrax T25 Dispersing Instrument (IKA) with a rotor–stator mechanism, and RNA was subsequently isolated using AllPrep DNA/RNA Mini-Kit (Qiagen) according to the manufacturer’s instructions. This included a DNase treatment step to remove potentially contaminating genomic DNA. RNA (600 ng) was reverse-transcribed to cDNA using SuperScript II RT (Invitrogen) before use in a quantitative real-time polymerase chain reaction (QPCR) using Platinum SYBR Green qPCR SuperMix-UDG (Invitrogen). Amplification and detection were performed in a 40-cycle reaction (primer annealing temperature: 58 °C) using Mx3000P according to the manufacturer’s instructions (Stratagene). This included the generation of a melt curve at the end of each QPCR run to ensure single product formation. Gene-specific primers used are listed in Table 1. GAPDH was used as an internal control to normalize gene expression between samples. The samples were quantified using the delta Ct method and log-transformed to normalise.

**Table 1:** Primer sequences.

Gene	Forward Primer	Reverse Primer
GAPDH	ACAGTCAGCCGCATCTTCTT	ACGACCAAATCCGTTGACTC
Collagen 1 (Col1A1)	GAACGCGTGTCAATCCCTTGT	GAACGAGGTAGTCTTTCAGCAACA
Collagen 3 (Col3A1)	AACACGCAAGGCTGTGAGACT	GCCAACGTCCACACCAAATT
Galectin-3	GCAGACAATTTTTCGCTCCAT	GATAGGAAGCCCCTGGGTAG
MMP2	CCACGTGACAAGCCCATGGGGCCCC	GCAGCCTAGCCAGTCGGATTTGATG
MMP9	GTGCTGGGCTGCTGCTTTGCTG	GTCGCCCTCAAAGGTTTGGAAT
Tetranectin	CCTTCACCCAGACGAAGACC	CGCAGGTACTCATACAGGGC
TIMP1	CTTCTGGCATCCTGTTGTTG	AGAAGGCCGTCTGTGGGT

#### Histological tissue staining

Tissues were formalin-fixed and paraffin-embedded, cut into 8 µm thick sections, and stored at 4 °C until used in histological immunostaining. Before staining, sections were deparaffinised and rehydrated using a Leica Autostainer XL, incubated in citrate buffer antigen retrieval solution (10 mM Citric Acid, 0.05% Tween 0.5%, pH 6.0), and blocked (protein block, Dako). Sections were subsequently stained for 1 h with either a rabbit anti-human monoclonal antibody against Tetranectin (Abcam), or a mouse anti-human monoclonal antibody against CD68 (Dako), or for 2 h with Picrosirius red solution (0.01% Direct red 80 and 0.01% Fast green diluted in Picric acid aqueous solution (Sigma)) for histological visualization of collagen 1 and 3 fibers, i.e. collagen deposition within the cardiac tissue. For Tetranectin staining, the Dako Real Envision detection system, Peroxidase/DAB + , rabbit/mouse (Dako) was used according to the manufacture’s instruction. For CD68 staining, a non-HRP, anti-mouse alkaline phosphatase secondary antibody (Abcam) was used. Stained sections were counterstained with Haematoxylin using Leica Autostainer XL and imaged at 40-fold magnification using Aperio ScanScope digital scanner and VisionScope V10 (Aperio Technologies). A positive pixel count algorithm was applied to analyse the area and intensity of the stain. For picrosirius red staining, sections were rinsed in water, dehydrated with increasing concentrations of alcohol using Leica Autostainer XL and imaged also at 40-fold magnification. For Tetranectin/picrosirius red and Tetranectin/CD68 double stainings, sections were first stained for Tetranectin and then counterstained with either picrosirius red or CD68 as described above.

### Statistical analysis

For continuous variables, summary statistics are presented as the mean ± standard deviation (SD) or median and 25–75^th^ percentiles. Categorical variables are presented as frequencies and percentages (in parenthesis). Comparisons between the low- and high-Tetranectin groups were made using independent t-test or Mann-Whitney test where appropriate. The relationships between Tetranectin, markers of collagen turnover and markers of inflammation were assessed using Pearson or Spearman correlation coefficients for variables that were normally or non-normally distributed. A p-value of less than 0.05 was considered statistically significant.

## Results

### Circulating levels of Tetranectin in peripheral serum in patients with HF compared to those patients without HF

**Figure 1:**
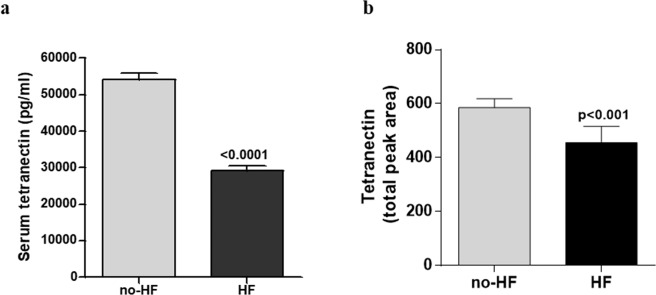
Circulating serum Tetranectin levels in patients without (n = 60) and with (n = 40) heart failure (HF) from study cohort 1, validation cohort, as identified by ELISA (**a**), and in patients without (n = 164) and with (n = 60) HF from study cohort 2, re-validation cohort, as identified by targeted mass spectrometry method Selected Reaction Monitoring (SRM) (**b**). Data represent mean ± SD and analysis performed using independent t-test or Mann-Whitney test.

To confirm previous proteomics findings^6^, the expression of serum Tetranectin was quantified in a validation cohort (study cohort 1), consisting of 100 patients visiting the heart failure and STOP-HF clinics: 60 no-HF patients and 40 symptomatic HF patients. Patient characteristics of study cohort 1 are described in Table 2. Results indicate that circulating levels of Tetranectin is reduced by approximately 50% in HF patients compare with asymptomatic controls (Fig. 1a, p < 0.0001).

**Table 2:** Patient characteristics of study population 1.

Variable	no-HF (n = 60)	HF (n = 40)
Age, years	70 ± 8	72 ± 13
Gender, male	30 (50%)	20 (50%)
SBP, mmHg	142 ± 16	133 ± 21^*^
BNP, pg/ml	23 (13:36)	107 (45:231)^***^
Diabetes Mellitus	8 (13%)	11 (28%)
Hypertension	42 (70%)	18 (45%)^*^
**Medications**		
RAAS Inhibitor	32 (53%)	35 (88%)^***^
Βeta-Blocker	16 (27%)	30 (75%)^***^
Statin	42 (70%)	20 (50%)^*^
Diuretic	21 (35%)	30 (75%)^***^
**Echocardiography**		
EF, %	68 ± 9	60 ± 8^***^
E/E’	8.8 ± 3.1	9.6 ± 3.5
LVMI, g/m^2^	97 ± 23	127 ± 37^***^
LAVI, mls/m^2^	23 ± 4	46 ± 2^***^

Values are mean ± SD, mean (25th:75th percentiles) or n (%). SBP, systolic blood pressure; RAAS Inhibitor, renin angiotensin system inhibitor; EF, ejection fraction; LVMI, left ventricular mass index; LAVI, left atrial volume index. P-value <0.05 is significant and depicted with *p < 0.01 – with **; and p < 0.001 – with ***.

Re-validation of reduced Tetranectin levels was achieved in a second, independent cohort of 224 patients with risk factors (hypertension and/or diabetes) for heart failure (n = 164) or with heart failure (n = 60) using a mass spectrometry approach, designed to target the specific Tetranectin peptide fragment LDTLAQEVALLK. Patient characteristics of the study cohort are included in Supplementary Table 1. Circulating Tetranectin levels are significantly reduced in heart failure patients from study cohort 2 (Fig. 1b, p < 0.001).

### HF diagnosis with serum Tetranectin and BNP

**Figure 2:**
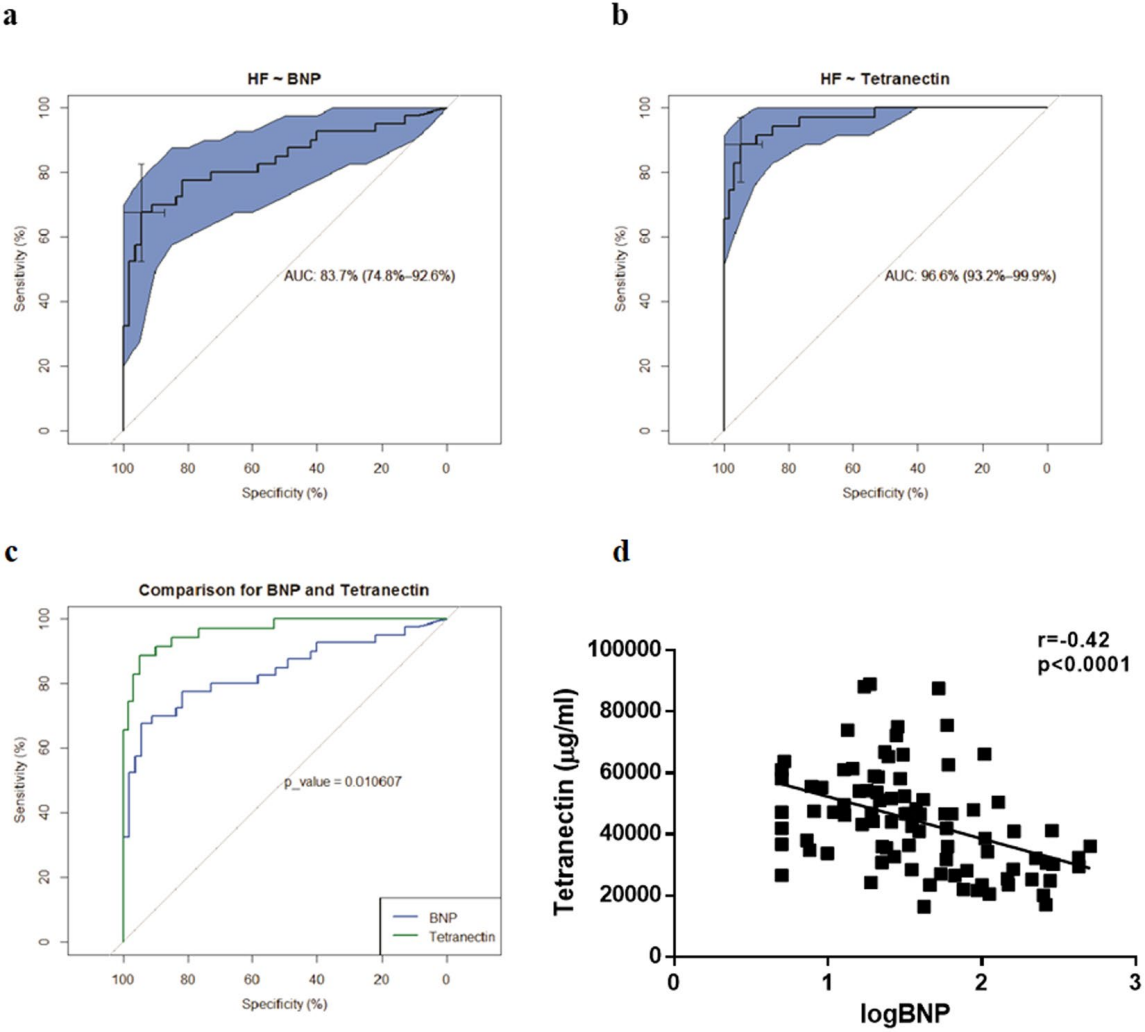
ROC curves for Tetranectin (**a**), BNP (**b**), and a comparison of Tetranectin and BNP (**c**) differentiating between patients without and with HF (study cohort 1, n = 100). Circulating Tetranectin and BNP were measured using ELISA and Triage Meter Point of care assay, respectively. Specificity and sensitivity thresholds, and area under the curve (AUC) with 95% Confidence Intervals presented. Correlation between Tetranectin and BNP is highlighted (**d**). Correlation coefficient was assessed using Pearson method.

Area under the receiver operating characteristic (ROC) curve (AUC) for the diagnosis of HF in the validation cohort of patients without and with HF (study cohort 1, n = 100) demonstrated a significantly higher diagnostic specificity and sensitivity of serum Tetranectin in univariate analysis (AUC = 96.6% [93.2–99.9%]) compared to the gold-standard marker BNP (AUC = 83.7% [74.8–92.6%]), p = 0.011, Fig. 2. In addition, BNP performance characteristics for HF diagnosis was significantly improved with addition of Tetranectin, p = 0.012. Tetranectin performance characteristics for HF diagnosis was not significantly improved with addition of BNP, p = 0.48. Inclusion of age and sex did not improve any model. Although a moderate but significant negative correlation between BNP and Tetranectin exists (r = −0.42, p < 0.0001, Fig. 2), using the ratio of the two circulating proteins does not increase the AUC compared with Tetranectin alone (AUC = 90.7% [83.2%-97.4%]).

### Correlation of serum Tetranectin levels with circulating markers of fibrosis and inflammation

**Figure 3:**
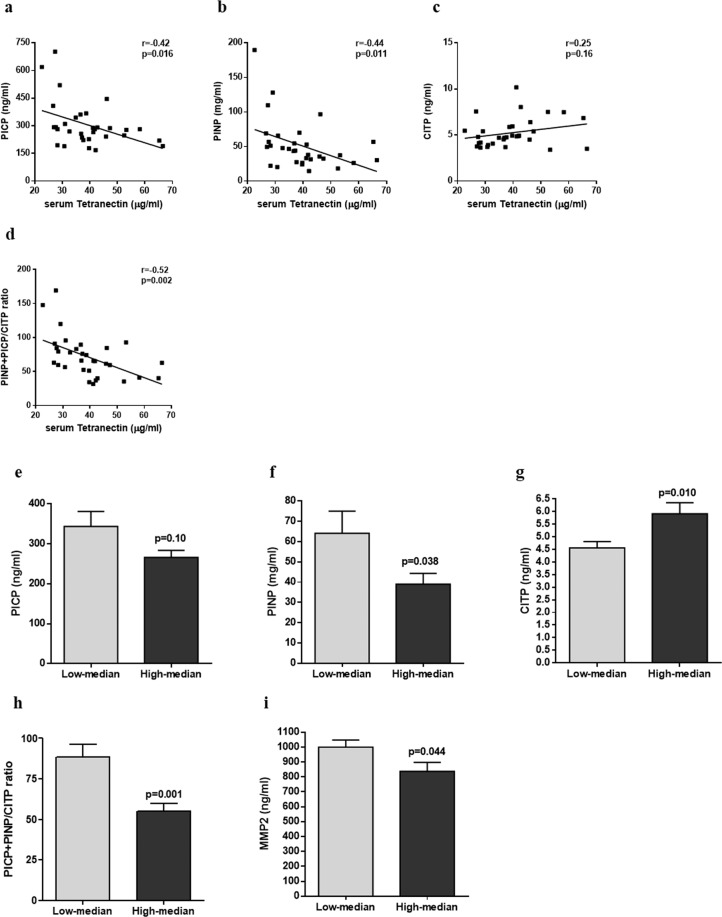
The relationship between serum Tetranectin levels with circulating markers of fibrosis – PICP (**a**), PINP (**b**), CITP (**c**), and PINP + PICP/CITP ratio (**d**), were assessed using correlations and Pearson or Spearman correlation coefficients; and by stratifying the patient population based on the median serum Tetranectin levels, PICP (e), PINP (f), CITP (g), and PINP + PICP/CITP ratio (h), and MMP2 (i). Circulating fibrosis markers were quantified using ELISA and radioimmunoassy. Cohort size, n = 32. Data represent mean ± SD and analysis performed using independent t-test or Mann-Whitney test.

Serum from study cohort 3 (cardiac surgery patients; characteristics are highlighted in Table 3) was utilised to investigate the relationship of serum Tetranectin levels with circulating levels of known markers of fibrosis and inflammation. Significant negative correlations with Tetranectin were observed with the products of collagen I synthesis PINP (r = -0.44, p = 0.011) and PICP (r = -0.42, p = 0.016), a modest positive correlation trend was observed with the product of collagen I degradation CITP (r = 0.25, p = 0.16), and a significant negative correlation was seen with the overall index of collagen I turnover PINP + PICP/CITP (r = -0.52, p = 0.002) (Fig. 3a). In addition, significantly lower levels of PINP, MMP2 and PINP + PICP/CITP collagen I ratio, and significantly higher CITP levels were associated with the high-median Tetranectin group upon separation of the cohort according to median serum Tetranectin levels (16 vs. 16 patients, Fig. 3b). Inflammatory markers, including TNFα, IL6, IL8, hsCRP, and MCP1 showed no significant correlation to serum Tetranectin.

**Table 3:** Patient characteristics of study population 3: the cardiac tissue cohort.

Variable	Value
Age, years	68 ± 10
Gender, male	24 (75%)
SBP, mmHg	136 ± 7
DBP, mmHg	81 ± 7
BMI, kg/m^2^	27 ± 3
BNP, pg/ml	40 (15:109)
**Medical history**	
Atrial Fibrillation	8 (25%)
Diabetes Mellitus	6 (19%)
Smoking History	11 (33%)
Hypercholesterolemia	8 (25%)
Ischemic Heart Disease	23 (70%)
Valvular Heart Disease	16 (50%)
Hypertension	12 (38%)
**Medications**	
RAAS Inhibitor	15 (47%)
Βeta-Blocker	21 (64%)
Statin	18 (56%)
Loop diuretic	12 (38%)
Calcium channel blocker	4 (13%)
**Echocardiography**	
EF, %	57 ± 7
LVIDd, mm	52.6 ± 5.7
IVS, mm	9.7 ± 1.5
PW, mm	10.9 ± 1.7
E/e’	9.3 ± 2.6
LAVI, mls/m^2^	28.8 ± 3.6

Values are mean ± SD, mean (25th:75th percentiles) or n (%). SBP/DBP, systolic and diastolic blood pressure; BMI, body mass index; RAAS Inhibitor, renin angiotensin system inhibitor; EF, ejection fraction; LVIDd, left ventricular end diastolic dimension; IVS, intraventricular septum; PW, posterior wall; E/e′, ratio of mitral early diastolic flow velocity over tissue Doppler lateral mitral annular lengthening velocity; LAVI, left atrial volume index.

### Correlation of cardiac tissue Tetranectin gene expression with tissue expression of fibrotic genes

**Figure 4:**
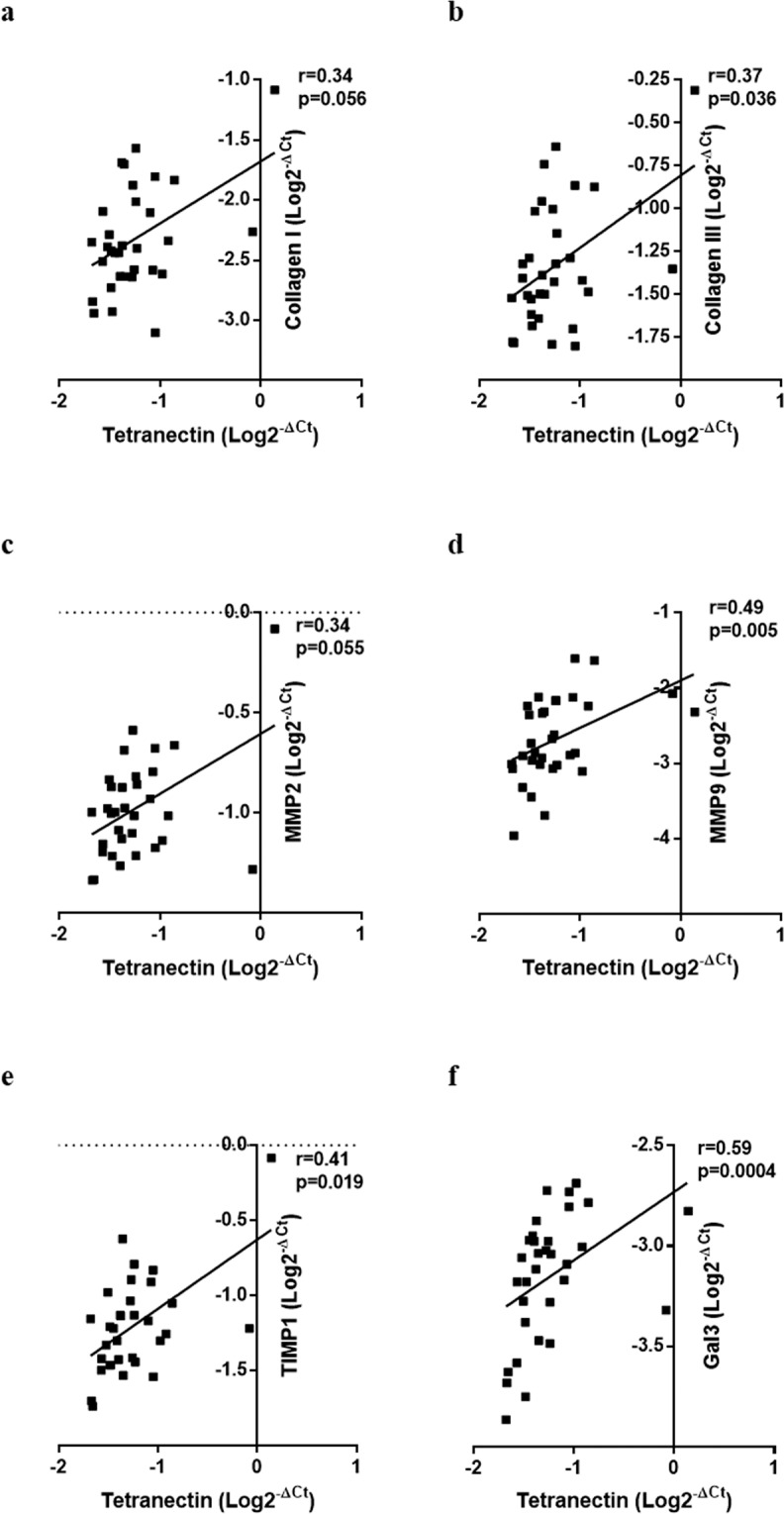
Correlation of Tetranectin tissue gene expression with expression of collagen I (**a**), collagen III (**b**), MMP2 (**c**), MMP9 (**d**), TIMP1 (**e**), and galectin-3 (**f**) tissue genes in human cardiac samples obtained from n = 32 cardiac by-pass patients. Gene expression was assessed using quantitative real-time PCR. Correlation coefficient was assessed using Pearson or Spearman method.

Within the human atrial appendage tissues, Tetranectin gene expression was significantly positively correlated to tissue gene expression of collagen 1 (r = 0.34, p = 0.056) and 3 (r = 0.37, p = 0.036), MMP2 (r = 0.34, p = 0.055), MMP9 (r = 0.49, p = 0.005), and TIMP1 (r = 0.41, p = 0.019) (Fig. 4). In addition Tetranectin gene was associated with gene expression of the cardiac fibrosis and heart failure biomarker galectin-3 (r = 0.59, p = 0.0004).

### Cardiac tissue Tetranectin protein association with collagen levels

**Figure 5:**
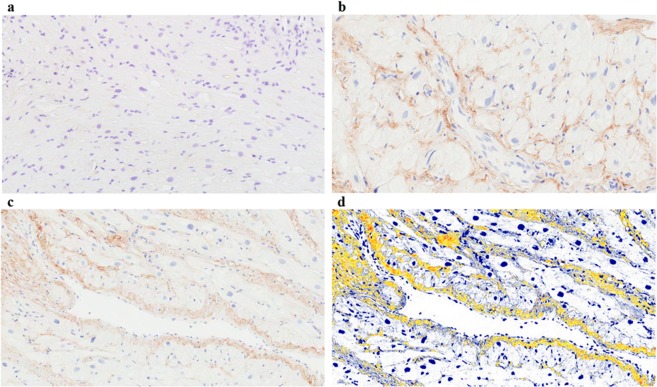
Tetranectin immunohistochemical tissue staining. Human cardiac tissue was obtained from cardiac by-pass patients, formalin-fixed and paraffin-embedded for immunostaining with either a rabbit anti-human monoclonal antibody against Tetranectin or an IgG isotype control antibody. Sections were counterstained with Haematoxylin and imaged using Aperio ScanScope digital scanner. A positive pixel count algorithm was applied to analyse the area and intensity of immunoreactivity. Representative images presented; IgG control (**a**); examples of Tetranectin (brown) and haematoxylin (blue) stained tissue sections (**b**,**c**); Example of the Tetranectin positive pixel algorithm used to quantify tissue protein expression (**d**). Images were captured at 20x magnification.

**Figure 6:**
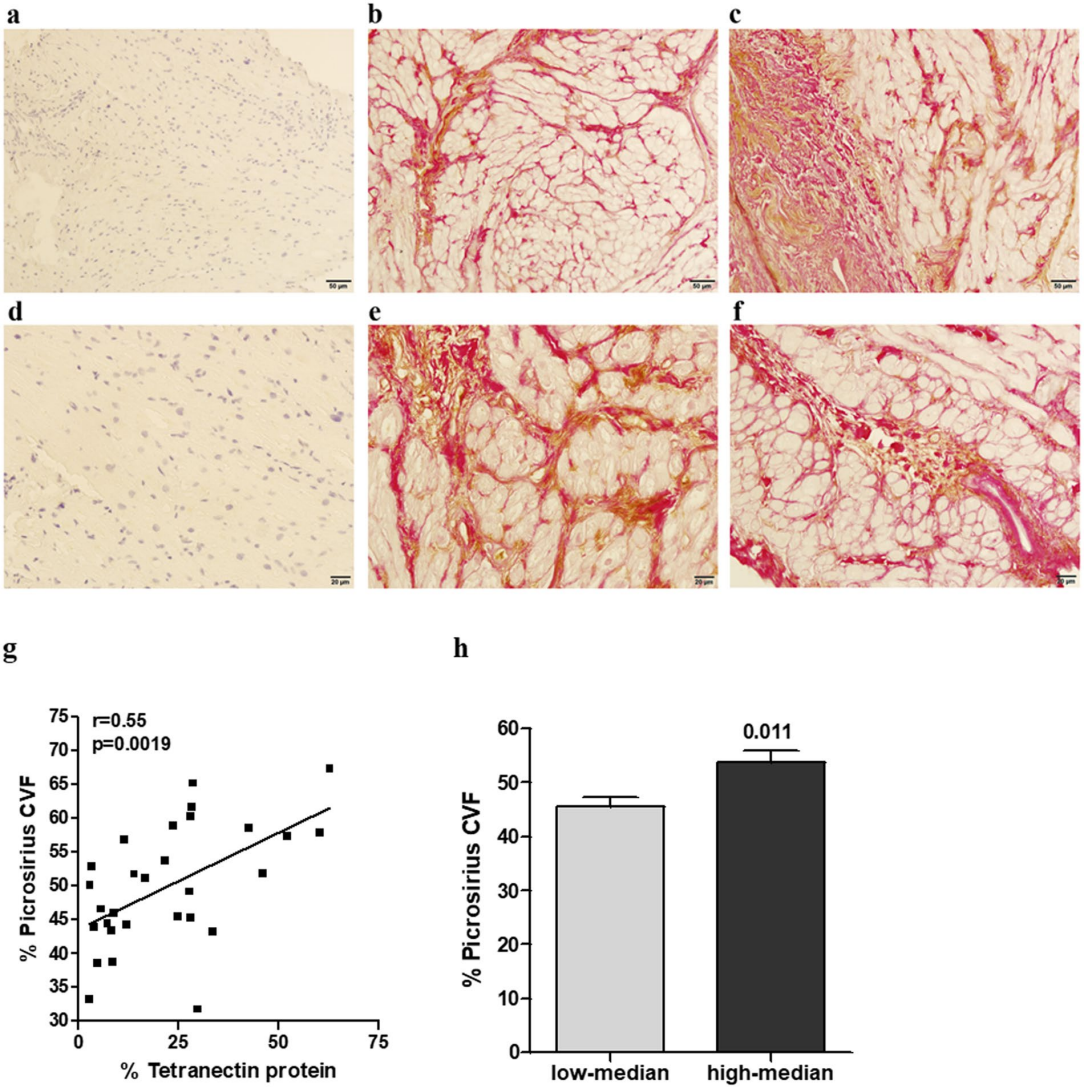
Localization of Tetranectin and collagen in human cardiac tissue sections obtained from n = 32 cardiac by-pass patients. Tissue was formalin-fixed and paraffin-embedded for immunostaining with either a rabbit anti-human monoclonal antibody against Tetranectin or an IgG isotype control antibody. Picrosirius red staining was used to assess collagen. Sections were counterstained with Haematoxylin and imaged using Aperio ScanScope digital scanner. A positive pixel count algorithm was applied to analyse the area and intensity of immunoreactivity. Representative images presented, from left to right: IgG control and haematoxylin (blue) stained tissue sections (**a**) and two examples of Tetranectin (brown) and Picrosirius collagen (red) immunohistochemical tissue dual staining in cardiac tissue at 20× (**a**–**c**), and 40-fold magnification (**d**–**f**). Correlation of Tetranectin protein expression (%) with picrosirius collagen (% collagen volume fraction, CVF), (**g**). Correlation coefficient was assessed using Pearson method. Collagen deposition (CVF) in patients with high and low tissue levels of Tetranectin (patients stratified by median Tetranectin levels), (**h**). Data presented as the mean ± SD and analysis performed using independent t-test.

Expression of Tetranectin protein was detected within human atrial appendage tissue by immunohistochemistry (Fig. 5). Tetranectin is visible in interstitial and perivascular regions. Localization of Tetranectin and fibrotic regions was assessed using dual immunohistochemical staining of Tetranectin and collagen (Picrosirius red) (Fig. 6). Quantification (positive pixel analysis) of Tetranectin and collagen (Picrosirius red) tissue stains showed significant positive correlation between Tetranectin positivity and total collagen content (r = 0.55, p = 0.0019, Fig. 6c). In addition, significantly increased levels of collagen was identified in cardiac tissue of patients with high Tetranectin protein expression (p = 0.011, Fig. 6d).

### Immunohistochemical analysis of inflammatory cells and Tetranectin

Tetranectin and CD68-macrophage dual staining in atrial appendage tissues reveals macrophage localization predominantly to perivascular areas and dense fibrous tissue areas. Tetranectin appears in the interstitium, perivascular areas, and dense fibrous tissue regions. CD68-positive cells did not co-stain for intracellular Tetranectin in this tissue (Supplementary Fig. 1).

## Discussion

We report for the first time on human cardiac tissue based expression of Tetranectin (beyond coronary artery expression). In two cohorts totalling 324 patients with either risk factors for heart failure (hypertension and/or diabetes) or with heart failure, we demonstrate that circulating levels of Tetranectin are significantly decreased in those with HF. Serum Tetranectin demonstrated higher diagnostic specificity and sensitivity for heart failure than the gold-standard biomarker BNP. Indeed, a combination of Tetranectin with BNP achieved high performance characteristics for HF diagnosis (not further improved by controlling for age and gender). Although circulating serum Tetranectin levels correlated negatively with circulating markers of fibrosis, an opposite relationship was found within cardiac tissue of 32 patients with ischemic or valvular heart disease, where expression levels correlated positively with fibrosis and fibrotic genes.

Within the literature, serum concentrations of Tetranectin have been shown to be reduced in non-cardiac pathological conditions (cancer, inflammatory diseases), possibly indicating a common underlying disease process among these diseases. However, none of these studies have compared circulating vs. organ-specific tissue levels to provide a direct link of Tetranectin with a certain disease pathology. It would be of great interest to investigate this to see if a similar inverse pattern is observed in other diseases. Relevant to the biomarker potential of Tetranectin for heart failure, combining Tetranectin with other novel heart failure biomarkers may improve disease specificity in a multi-morbid condition.

From a cardiovascular perspective, prior literature has demonstrated an anti-thrombotic role (via enhanced plasminogen activation) and an anti-proliferative role (regarding the endothelium) for platelet-released Tetranectin^24^. Higher plasma Tetranectin levels were inversely associated with risk of atherosclerotic cardiovascular disease^16^. Chen *et al*. previously reported lower serum Tetranectin levels in patients with coronary artery disease (proportional to the disease burden) compared to healthy subjects and hypothesized that atherosclerosis-related endothelial damage might lead to intimal accumulation of Tetranectin in atherosclerotic plaque complexes with Lipoprotein (a) and/or fibrin, thus diminishing serum Tetranectin levels^25^. Of note, they performed immuno-histochemical staining for Tetranectin in human normal internal mammary artery and atherosclerotic coronary artery and found significantly higher expression in patients with coronary artery disease compared with healthy controls. The model proposed by Weber *et al*. for the development of interstitial fibrosis includes pathophysiological processes such as endothelial damage (inducing hormonally mediated coronary hyperpermeability) as an inciting factor, not distinct from atherosclerosis^26^. In this study Tetranectin was found to co-localise with areas of collagen deposition and we hypothesize that reduced Tetranectin levels in the circulation could either indicate cardiac uptake to help combat myocardial interstitial fibrosis or that perhaps reduced circulating Tetranectin might predispose to the development of heart failure. More recently, a 2018 biomarker analysis of the Framingham cohort in the US has shown that reduced circulating levels of Tetranectin (CLEC3B) are significantly associated with all‐cause mortality and with cardiovascular disease death^27^. In further support of a role for Tetranectin in heart disease, a small study (n = 10 per group) was published last year reporting than circulating levels are significantly reduced within three hours following an acute myocardial infarction^28^. All of these scenarios would point towards a cardioprotective role for Tetranectin and require further investigation.

In conclusion, we report for the first time that Tetranectin is a promising candidate HF biomarker associated with fibrotic processes within the myocardium. Further investigations into the mechanisms and consequences for altered circulatory and cardiac tissue Tetranectin are needed. In addition, it is important to expand our novel biomarker findings into other independent heart failure cohorts across a range of geographical locations, and to compare the diagnostic and clinical value with existing heart failure biomarkers and cardiac imaging data, as well as investigate the dynamic response of Tetranectin to change in cardiac status in a longitudinal study.

## Limitations

Although this is the first study to report on Tetranectin as a potential heart failure biomarker, these observations were made on a relatively small cohort, and validation studies in additional representative screening populations harbouring multiple different diagnoses, such as cardiomyopathy and acute decompensated HF (ADHF), would help confirm the cardiac specificity of Tetranectin as a prospective biomarker. BNP may also perform better in the acute HF setting. Given the data presented further research in this area would be of value. The tissue analysis conducted in this study was on human cardiac samples, however, these were derived from atrial samples from patients undergoing cardiothoracic surgery. This human cardiac tissue served as an *ex vivo* model by which to assess for tissue specific associations of Tetranectin with fibrosis and inflammation. Further work would be required to examine Tetranectin in this context from ventricular tissue, however access to this material in humans is challenging.

## Supplementary information

Supplemental File.
