# Supplementary material for: Tetranectin, a potential novel diagnostic biomarker of heart failure, is expressed within the myocardium and associates with cardiac fibrosis
Source: Sci Rep. 2020 May 5;10:7507. doi: 10.1038/s41598-020-64558-4 (PMC7200823; doi:10.1038/s41598-020-64558-4)

Tetranectin, a potential novel diagnostic biomarker of heart failure, is expressed within the myocardium and associates with cardiac fibrosis

Kenneth McDonald, MD [1,†], Nadezhda Glezeva, PhD [1, 2,†], Patrick Collier, PhD [3], James O'Reilly, BSc [2], Eoin O'Connell, MLitt [1], Isaac Tea, MD [2], Adam Russell-Hallinan, PhD [2, 4], Claire Tonry [2,4], Steve Pennington [2], Joe Gallagher, MD [1], Mark Ledwidge, PhD [1], John Baugh, PhD [2,†], Chris J. Watson, PhD [1,2,4, †,*]

[1] Heart Failure Unit, St Vincent's University Hospital Healthcare Group, Elm Park, Dublin, Ireland

[2] Conway Institute of Biomolecular and Biomedical Research, School of Medicine, University College Dublin, Ireland

[3] Department of Cardiovascular Medicine, Cleveland Clinic, Cleveland, Ohio 44195, USA

[4] Wellcome Wolfson Institute for Experimental Medicine, Queen's University Belfast, Northern Ireland, UK

[†]Equal contribution

* Corresponding author

Supplementary Methods

SRM Assay Design

The SwissProt accession number for Tetranectin was used to conduct a search for proteotypic peptides suitable for SRM analysis using Skyline (MacCoss laboratory, Washington DC version 1.4) and Spectrum Mill Peptide Selector (Agilent Technologies, version 3.3.078). Peptides were selected based on the following criteria; no missed cleavages in the peptide sequence, sequence length of 8-25 amino acids (AA), no potential ragged ends and no reactive Cysteine (C) or Methionine (M) residues within the sequence (these AA are susceptible to covalent modifications which would affect the peptide's m/z). Each peptide also had to match either an *in-house* or pubic spectral library and qualify as being 'unique' (determined by the Skyline 'Uniqueness' search function). A review of recent literature based on SRM experiments also allowed for identification of relevant peptides for which SRM assays had already been developed. Where possible, peptides which were commonly identified in both Skyline and Peptide Selector and/or recent literature were selected. In cases where there was no overlap, the highest ranking peptides identified by Skyline were chosen for development. The charge state of precursor ions was set to 2 or 3 and the product ions were limited to singly charged y ions. In order to minimize potential interferences, y ions with m/z close to the precursor ion were excluded. No fewer than 2 peptides were selected for each protein. Five transitions per peptide with the highest MS signals in the available MS spectral libraries were used for the initial SRM development. To aid in the development of SRM assays synthetic crude peptides were obtained from Thermo Fisher Scientific [PEPotecTM SRM unmodified peptide in plate]. Once received, synthetic peptides were pooled together to a final concentration of 200pmol. The synthetic peptide pool was analysed in an Agilent 6460 Q-Tof mass spectrometer. Spectra required for SRM assay design in Skyline were built using Trans Protemic Pipeline (TPP) software. Agilent .d files resulting from analysis of the peptide pool on the Q-Tof were first converted to mxXML format using MS Convert. Files were searched against a Uniprot Human database (Release 01-2011) using the X Tandem search engine to generate a combined pep.xml data file ready for import and spectral library building in Skyline. A probability score cut-off of 0.9 was applied to all spectral libraries generated in skyline. The spectral library data was used to build a transition list inclusive of the synthetic crude peptides, with five transitions per peptide as before. To determine the ability of the QqQ to detect Tetranectin within a serum matrix, the synthetic peptide pool was spiked into serum samples at a ratio of 1:5 and analysed on the QqQ.

SRM Analysis

SRM analysis was carried out on a nanoflow reverse phase C18 chromatographic Chip Cube based separation, coupled to a 6460 triple quadrupole mass spectrometer (QqQ). 100µg of trypsin-digested crude and depleted serum samples were reconstituted in 100µl Buffer A [3% ACN 0.1% FA (H20)] and centrifuged for 30 min at 4°C. Samples were analysed using a 40 minute separation LC run going from 0 - 95% Buffer B [10% H20 0.1% formic acid (ACN)] along the following gradient; 0min 0%, 5 min 10%, 35 min 30%, 37 min 95%, 38 min 95% 40 min 0% (Figure 2.2). Dwell time was set to 10ms and Collision Energy was set by the following equation for each transition [(precursor ion/100)*3.6-4.8]. The accelerator voltage was maintained at 4. For both crude and depleted serum, 2µg of sample was loaded onto the HPLC chip at a flow rate of 3µl/min. Following enrichment on a trapping column (160nl), the peptides were separated on a C18 analytical column [Zorbax 3005B C18 5µm (150mm x 75µm)] before being passed into the QqQ through a nanospray needle also contained in the chip. Prior to the start of the SRM runs, 5µl of PepMix was loaded under the same experimental conditions with an 18 minute gradient [0 min 0%B, 9 min 35%B, 12 min 95% B, 15 min 95% B, 18 min 0% B (Figure 2.3)] as a means of assessing system suitability. In order to prevent carry over between samples, 'blanks' were loaded at the start and end of each SRM run and between each sample injection. In order to maintain reproducibility and confirm optimum instrument performance throughout the verification experiments on the 500 sample cohort, Pierce TM Peptide Retention Time Calibration Mixture (Thermo Scientific) was used as a 'quality control' (QC) (Table 2.1). This QC was loaded to a final concentration of 250fmol/µl at the beginning, middle and end of the SRM run in order to monitor and normalize QqQ performance between samples and over time.

Data analysis

Data analysis of all experiments described here was performed using both Qualitative Mass Hunter Software (Agilent, version) and Skyline (MacCoss lab). Qualitative Mass Hunter software was used for preliminary visual assessment of the results. Using this software, peak quality and signal intensity was recorded for each peptide as well as the quality of the overall total ion chromatogram (TIC) for each samples and the QC. An acceptable recording for signal strength was 10^3 or greater, while 10^1 indicated a very low signal. Analysis of the data using Skyline software provided information on dot product, ranking, retention time and correlation to spectral library data for each peptide and transition. All peaks were manually reviewed to ensure (based on dot product and RT) that the true representative peak for each measured peptide was being analysed. For statistical analysis of the results obtained for each of the patient samples, a custom report was generated in Skyline to include information on 'Peptide Sequence', 'Protein Name', 'Replicate Name', 'Library Rank, 'Peptide Retention Time', 'Library Dot Product' and 'Area'.

Supplementary Results

Table 1 Patient characteristics of study cohort 2: re-validation cohort

**Variable**	**no-HF (n=164)**	**HF (n=60)**
**Age, years**	67 ± 10	75 ± 7 ***
**Gender, male**	83 (51%)	35 (58%)
**SBP, mmHg**	134 ± 17	123 ± 22 ***
**Diabetes Mellitus**	22 (13%)	18 (30%) **
**Hypertension**	102 (62%)	48 (80%) *
**<u>Medications</u>**		
**RAAS Inhibitor**	51 (31%)	33 (55%) **
**Beta-Blocker**	52 (32%)	52 (87%) ***
**Statin**	125 (76%)	38 (63%)
**Diuretic**	45 (27%)	55 (92%) ***
**<u>Echocardiography</u>**		
**EF, %**	67 ± 8	61 ± 7 ***
**E/E'**	9.0 ± 3.3	11.2 ± 3.7 ***
**LVMI, g/m^2**	90 ± 23	116 ± 34 ***
**LAVI, mls/m^2**	27 ± 10	52 ± 19 ***

Values are mean ± SD, mean (25th:75th percentiles) or n (%). SBP, systolic blood pressure; RAAS Inhibitor, renin angiotensin system inhibitor; EF, ejection fraction; LVMI, left ventricular mass index; LAVI, left atrial volume index. P-value <0.05 is significant and depicted with *; p<0.01 – with **; and p<0.001 – with ***.

Figure 1 CD68 macrophage and Tetranectin immunohistochemical tissue staining in human cardiac tissue sections obtained from cardiac by-pass patients. A) CD68 macrophage single stain (red), B) Tetranectin single stain (brown), and C) CD68 marcrophage (red) and Tetranectin (brown) dual stain. Tissue sections are counter-stained with haematoxylin (blue). Images were scanned using Aperio ScanScope digital scanner. CD68 appears predominantly in perivascular areas and dense fibrous tissue areas. Tetranectin appears in interstitium, perivascular areas, and dense fibrous tissue regions populated with cardiac fibroblasts. Images were captured at 20x magnification.

A

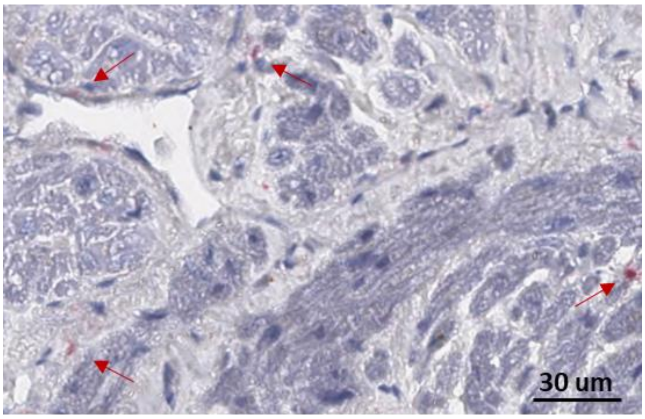

B

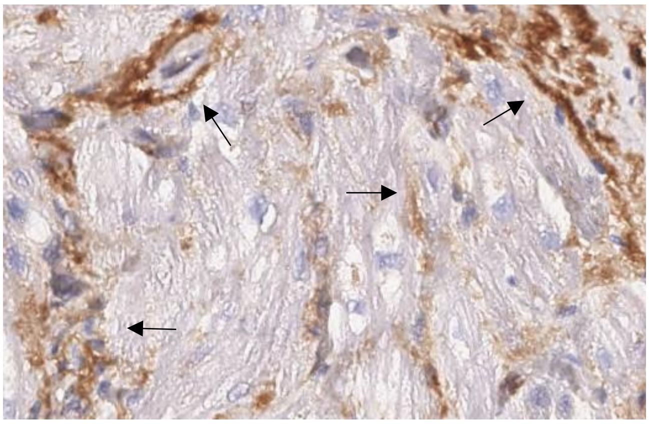

C

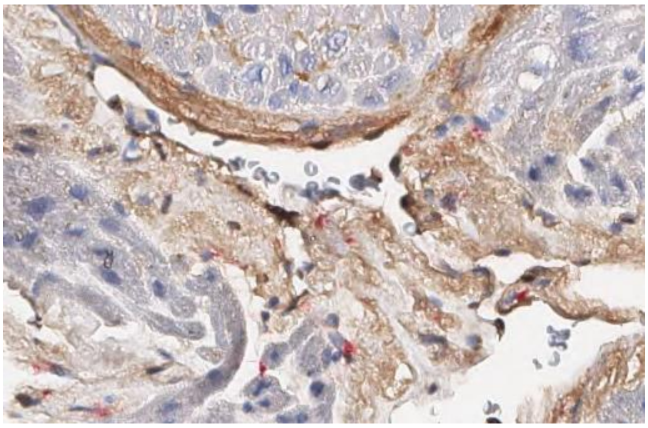

Supplement: Supplementary file 1 — Supplemental File. [file 41598_2020_64558_MOESM1_ESM.pdf]
